# Antenatal Dads and First Year Families program: a qualitative study of fathers’ and program facilitators’ experiences of a community-based program in Australia

**DOI:** 10.1017/S1463423619000768

**Published:** 2019-12-10

**Authors:** Yvonne Karen Parry, Matthew David Ankers, Shelly Abbott, Lyall Willis, Lynne Thorpe, Teresa O’Brien, Curtis Richards

**Affiliations:** 1 Senior lecturer, College of Nursing and Health Sciences, Flinders University, Bedford Park, South Australia, Australia; 2 PhD Candidate, College of Nursing and Health Sciences, Flinders University, Bedford Park, South Australia, Australia; 3 Associate Lecturer, College of Nursing and Health Sciences, Flinders University, Bedford Park, South Australia, Australia; 4 Senior Men’s and Families worker, Centacare Family Services, Murray Bridge, South Australia, Australia; 5 Manager, Centacare Family Services, Murray Bridge, South Australia, Australia; 6 Manager, Communities for Children Programs, ac.care Murraylands, Murray Bridge, South Australia, Australia; 7 Manager, ac.care Murraylands, Murray Bridge, South Australia, Australia

**Keywords:** community-based parenting programs, fatherhood and depression, fatherhood programs, fathers and postnatal depression, parenting and fathers

## Abstract

**Aim:**

Currently, there is limited knowledge on the impact of father-only sessions or parenting programs supporting impending fatherhood. This research explored an antenatal dads program aimed at fathers to assess the benefits of such interventions.

**Background:**

Literature regarding parenting programs and early childhood education initiatives, especially those aimed at children and families in disadvantaged circumstance, have been demonstrated to act as a buffer to poorer health and lifestyle outcomes in later life.

**Methods:**

A qualitative research approach was used to explore the experiences of 16 fathers and 6 staff of a community-based parenting program with sessions focusing on fatherhood.

**Findings:**

Four main themes were identified from the data regarding the experiences of groups engaged with the Antenatal Dads and First Year Families program. The first theme ‘Knowledge and Capacity Building’ stated that the information provided in the program helped fathers to be better informed and prepared for their impending fatherhood. The second theme was ‘Mental Health Awareness’ and identified the importance of raising awareness of depression and suicide in fathers, including where and how to get help. The third theme was ‘Soft-Entry’ and highlighted how the attendance at one service helped participants to learn about additional services through word of mouth and targeted promotion. The final theme was ‘Feeling Connected’, which helped fathers to feel more connected with the process of childbirth and development including playing and engaging with their children. Overall, the fathers found that the male-only sessions assisted them by supporting frank discussions on fatherhood. Additionally, the study helped identify the advantages of fathers meeting other fathers through attendance in the program, or even other couples in similar situations that helped fathers to feel less lonely regarding their situation.

## Introduction

Parenting programs and early childhood education initiatives, especially those aimed at children and families in disadvantaged circumstance, have been demonstrated to act as a buffer to poorer health and lifestyle outcomes in later life (Marmot, 2006; Baum, 2011; Panter-Brick *et al.*, 2014; MacDougall, 2017). Research indicates that the ongoing benefits of these initiatives for disadvantaged children include higher education attainment, greater participation in employment, and improved physical and mental health in adulthood; the ongoing benefits for the community include lower crime rates and less welfare dependence (Noble *et al.*, 2005; Belfield *et al.*, 2006; Mustard, 2006; Tully, 2009; Mustard, 2010; Bartik, 2011; Moffitt *et al.*, 2011; Reynolds *et al.*, 2011; Richter & Naicker, 2013; Kraus *et al.*, 2014; Englund *et al.*, 2015; Parry & Abbott, 2018). Internationally, interventions such as the High/Scope Perry intensive preschool program^1^ and the Child-Parent Centre Program^2^ have demonstrated these trends across multiples studies, in adult populations who took part in these interventions as children, over multiple decades (Belfield *et al.*, 2006; Heckman *et al.*, 2010; Reynolds *et al.*, 2011; Englund *et al.*, 2015).

The demonstrated link between educational interventions and better health outcomes indicates that good health is more than a biological absence of disease. Rather, a person’s health is also impacted by their social circumstance as outlined in the Social Determinants of Health (SDH). The SDH acknowledge that the social, socio-economic and political conditions in which a person lives, works, learns and grows can impact a person’s health (Marmot, 2006; Maggi *et al.*, 2010; Baum, 2011; Keleher & MacDougall, 2017). For example, the effects of disadvantage can commence in utero where malnutrition, caused by a lack of parental material resources, causes low birth weight in the child (Wadsworth & Butterworth, 2006; Maggi *et al.*, 2010; Baum, 2011; MacDougall, 2017; Parry & Abbott, 2018). Children born into disadvantage will generally demonstrate impacts to their physical, cognitive, emotional and social development as well as achieving lower levels of education, which all impact on future health (Wadsworth & Butterworth, 2006; Taylor *et al.*, 2008; Maggi *et al.*, 2010; Keleher & MacDougall, 2017; MacDougall, 2017). In contrast, people of affluence demonstrate better health outcomes across the lifespan, signifying that social circumstances and health are both linked and stratified (Baum, 2011; Keleher & MacDougall, 2017). Encouragingly, interventions targeted at the individual, and the structural drivers of the SDH are recognised as aiding children and families to cope with, or even move beyond, vulnerable social conditions (Wilkinson *et al.*, 2009; Maggi *et al.*, 2010; Solar & Irwin 2010; Shonkoff & Garner 2012; Pickett & Wilkinson, 2015; Keleher, 2017).

One type of intervention that attempts to address the SDHs is the programs targeting the parents of children at risk. Parenting programs generally attempt to improve parenting knowledge and encourage behaviour change that in turn improves the child’s behavioural habits and developmental outcomes (Tully, 2009; Richter & Naicker, 2013; Love *et al.*, 2016; Nyberg *et al.*, 2016; Parry & Abbott, 2018). Parenting programs also seek to aid parent’s mental health, stress and maladaptive parenting techniques to provide a more favourable environment for the child and improve family wellbeing (Tully, 2009; Richter & Naicker, 2013; Borrelli *et al.*, 2015; Love *et al.*, 2016; Parry & Abbott, 2018). Regardless of the approach, all interventions attempt to reduce, or even prevent, a child living in disadvantage from the impact of the multiple and complex circumstantial vulnerabilities that accumulate to produce poorer adult health outcomes (Noble-Carr, 2007; Keys, 2009; Tully, 2009; Dockery *et al.*, 2010; Gibson & Johnstone, 2010; Lynam *et al.*, 2010; Solar & Irwin, 2010; Nelson & Mann, 2011; Kilmer *et al.*, 2012; McCoy-Roth *et al.*, 2012; Zlotnick *et al.*, 2012; Coren *et al.*, 2013; Embleton *et al.*, 2013; Roos *et al.*, 2013; Kuehn, 2014).

Research regarding parenting interventions suggests that engaging fathers in parenting programs increases the success of parenting interventions, reduces child abuse and neglect (through education and re-engagement of the father, or the father acting as a protective factor against abusive/neglectful mothers), and improves longer term childhood outcomes (Bronte-Tinkew *et al.*, 2007; Tully, 2009; Fletcher *et al*, 2014; Panter-Brick *et al.*, 2014; Scourfield, 2014). However, multiple barriers both structural and behavioural exist that inhibit the father’s participation in services including:

- Mothers who withhold partnership information from service providers due to domestic violence, child support issues or break down of relations (Scourfield, 2014; Ferrell, 2013; Zanoni *et al.*, 2013).
- Policy, resources and service structures focused on mothers (Ferrell, 2013; Zanoni *et al.*, 2013; Panter-Brick *et al.*, 2014; Scourfield, 2014; Darwin *et al.*, 2017; Baldwin *et al.*, 2018; Burgess & Goldman, 2018; Parry & Abbott, 2018) with fathers also reporting feeling left out regarding maternity services and parenting education programs (Bronte-Tinkew *et al.*, 2007; Fletcher *et al.*, 2014; Darwin *et al.*, 2017; Baldwin *et al.*, 2018).
- Men not wanting to take the focus of services away from the mother (including those that checked on mental health), despite fathers reporting that they wanted to be more involved (Darwin *et al.*, 2017; Baldwin *et al.*, 2018).
- Services not distinguishing between genders in their reporting (Zanoni *et al.*, 2013; Fletcher *et al.*, 2014; Panter-Brick *et al.*, 2014) or collecting limited data regarding fathers in general (Burgess & Goldman, 2018).
- An inability to clearly communicate the benefits of engagement with programs to the fathers (Fletcher *et al.*, 2014; Panter-Brick *et al.*, 2014; Darwin *et al.*, 2017; Baldwin *et al.*, 2018; Parry & Abbott, 2018).
- An assumption that fathers were abusive, incompetent or disinterested in parenting irrespective of actual evidence (Zanoni *et al.*, 2013; Burgess & Goldman, 2018).

To help mitigate some of the effects of disadvantage on children, the Australian Government Department of Social Services developed the Communities for Children (CfC) initiative in 2004 (Muir *et al.*, 2010). The CfC initiative funds Non-Government Organisation who, in consultation with local stakeholders, develop and implement strategic plans. These strategic plans attempt to improve the coordination of local services, address recognised gaps in services and build capacity within the community for children aged 0–12 years and their families (Muir *et al.*, 2010). One demonstrated benefit of the CfC initiative has been increased engagement with vulnerable and hard to reach population groups including Aboriginal and Torres Strait Islanders, Culturally and Linguistically Diverse (CALD), single-parent households, parents with low education attainment, or in households where one or both adults are unemployed (Muir *et al.*, 2010; Parry & Abbott, 2018).

The Murraylands region of South Australia has been recognised as an area where children have a higher susceptibility to developmental issues (Australian Early Development Census 2015). Accordingly, the Murraylands was identified as 1 of 52 areas where the CfC initiative was implemented. The CfC facilitator in the Murraylands of South Australia is ac.care who partners with local service providers to address and deliver identified service needs of young children and their families. One initiative delivered by ac.care’s community partner, Centacare, is the ‘Antenatal Dads and First Year Families program’ which is provided at various sites in the Murraylands region. Centacare runs specific antenatal education classes for fathers (Antenatal Dads program). The Antenatal Dads program draws on successful evidence-based programs to inform its service delivery including:

1. ‘Bringing Up Great Kids’ (BUGK) which focuses on the subjects of attachment narratives and mindfulness training. Hunter and Meredith (2015) review of BUGK suggested increased mindfulness in participating parents and improved relationships between family members.
2. ‘Hey Dad!’ an Indigenous specific program designed to build confidence in parenting and communication skills between Indigenous men and their children (Parker, 2009a; Rossiter *et al.*, 2017). The program was found to increase parenting skills through positively viewed, parenting education (Parker, 2009a; Bowes & Grace, 2014)
3. ‘Tuning in to Kids’ program focuses on supportive and emotionally controlled parenting that has demonstrated a decrease in parents’ dismissal of children’s emotions and improved child behaviour (Havighurst *et al.*, 2009; Parker, 2009b; Havighurst *et al.*, 2015).

The Antenatal Dads program has a structured weekly schedule that is the same across sites and is updated annually based on updates in the listed programs. Programs facilitators are dads with tertiary qualifications, such as Bachelor of Social Work with extensive training and knowledge in the areas of Domestic Violence, Community Work and Cultural Safety. Furthermore, the principal program worker has received training in all three listed programs as well as having additional training including a Diploma in Counselling and Community Services. The program helps to connect fathers with parenting through activities that address knowledge and understanding in areas of infant communication, attachment, brain development, postnatal depression (signs and symptoms in mothers), birthing, cognitive and linguistic development of infants and communication with spouse/partners. The First Year Families program acts as a follow-up program to explore the needs and concerns of fathers in their first year of fatherhood.

The program also aids fathers to access other services through the identification of individual needs and referrals to relevant agencies within the broader community service sector. However, as acknowledged, significant barriers have existed previously that impact on a father’s ability to participate in services, meaning literature regarding the impact, success or value of such programs is limited (Bronte-Tinkew *et al.*, 2007; Fletcher *et al.*, 2014; Scourfield, 2014). Previous research that has included the father often did not distinguish between genders or did not specifically explore the father’s views and experiences of parenting programs (Fletcher *et al.*, 2014; Panter-Brick *et al.*, 2014). The aim of this study was to use qualitative methods to explore the experiences of fathers who participated in the Antenatal Dads and First Year Families program to address this limitation within the literature. Furthermore, the study was an opportunity to understand the subjectively reported benefits for fathers who attended the program. Additional interviews and focus groups with service providers and their staff were also conducted to understand the broader context of the program and its perceived impact.

## Methodology

This investigation used a qualitative approach to explore the experiences of fathers undertaking the program, the experiences of staff delivering programs and the experiences of service providers and managers who organised and implemented programs. Initially, documentation from the CfC Murraylands program including performance analysis and quality improvement records was reviewed to provide background and insight to qualitative data collection. Using multiple sources of information to inform the study improved the studies rigour (Green & Thorogood, 2014; Patton, 2015). The next step involved consulting with managers and staff to help identify and produce a potential list of fathers who had been the recent recipients of the program (to help reduce recall bias), for inclusion in the research. Hansen (2006) describes this as a snowballing approach where key informants help the researcher identify participants with experience of the phenomena of interest. Patton (2015:46) relates choosing participants with experience of the research subject as a purposeful approach where participants are chosen on the basis of being ‘information rich’ regarding the phenomena of interest.

Fathers from the list were contacted by the research team with a letter of recruitment and a request to participate in the research. The research team consisted of three external researchers from Flinders University (R1, R2 and R3) experienced in the design, delivery and evaluation of health in the areas that include acute care, community setting, community parenting and child intervention programs. Fathers then self-selected to participate by contacting the research team to express their interest. This approach reduced coercion and helped ensure that program staff were unaware of those fathers who agreed to participate, which improved anonymity for participating fathers. Of the potential 29 fathers contacted, 16 volunteered to participate. Program facilitators (providers, managers and staff) were contacted via email with a letter of recruitment and a request to participate in the research. Program facilitators self-selected to participate by contacting the research team. The inclusion of program facilitators drew on a purposeful sampling approach as they were able to provide a different perspective of the same shared phenomena (the dad’s experience of the program) that helped give depth to the data (Patton, 2015).

Data were collected through two means including interviews and focus groups with providers, staff and parents. Interviews and focus groups drew on the same open question guide for continuity of results. The open question guide was devised from the reviewed literature and with consideration of the Antenatal Dads program framework that draws from the evidence-based programs outlined in the introduction. Additionally, a reference group consisting of members who delivered CfC programs assessed and offered advice on the open question guide, which included discussion on:

- the type of program;
- the usefulness of the program;
- the impact of the program on other aspects of the participants lives (e.g., the SDH);
- implications for changes;
- impact on health (mental and physical).

The initial data collection took place in the Murraylands region of rural South Australia in 2016. In total, 16 fathers and 6 service provider staff took part in the research. Table 1 illustrates the number of participants involved in each phase of data collection, notes the method of data collection and shows the basis for recruitment.

**Table 1.** The type of participants and method of data collection used

Participant type	Number	Basis for recruitment	Component of research (interview/focus group)
Program facilitators (managers and staff)	3	Responsible for delivery of the CfC programs	Focus group interview. Staff also provided comment on behavioural changes in fathers, families and child interactions and took part in follow-up clarification interviews
Community partners	3	Other service providers who refer to or, receive referrals from the program	Face-to-face interviews
Fathers (first-time and experienced fathers)	9	Participated in the Antenatal Dads and First Year Families program	Face-to-face or phone interviews
Fathers (first-time and experienced fathers)	7	Participated in the Antenatal Dads and First Year Families program	Focus group
Total participants	22		

CfC = Communities for Children.

Fathers were asked if they preferred to participate in a one-on-one interview or focus group. Written consent was gained before each interview and focus group from each participant. Two members of the research team collected the interview and focus group data (R1 and R2), two members conducted data analysis and coding together (R1 and R3), R2 conducted analysis and coding separately. The interviews ranged from 45 to 120 min in length, and the focus groups lasted up to 240 min in length. The recordings were transcribed verbatim by a third-party transcription service that has a confidentiality agreement with Flinders University, and pseudonyms were assigned to participants during the transcription phase to help maintain confidentiality. All participants were offered the opportunity to review their transcript, except those participants in the focus group, which was explained to the focus group participants via the letter of recruitment and prior to their involvement (Tong *et al.*, 2007).

Transcripts were manually coded (by R1 and R3); the first step involved reading the transcripts in full and noting participants’ responses of interest in text. This process was repeated and similar ideas were grouped together. This method of analysis is described by Patton (2015) as inductive where patterns and themes emerge from the data, rather than being imposed on them. Themes that emerged from the data were also analysed using a constant comparative method as described by Glaser and Strauss (1967). The constant comparative method compares the content of emerging themes against one another to ensure that theme content is relevant, and for alternative meaning of content that might connect content to other themes or indicate new ones (Glaser & Strauss, 1967; Cavana *et al.*, 2001; Tong *et al.*, 2007; Patton 2015). This way, care was taken in the review of statements and the development of themes to make them unique and to cover the range of possible meanings expressed in the data (Glaser & Strauss, 1967; Cavana *et al.*, 2001; Patton, 2015). Furthermore, this process helped identify statements that might not be core to the focus of study but remained important for the context and insight they provided to other statements made by participants. The findings were further validated by the use of a second coder (R2) (Green & Thorogood, 2014; Patton, 2015). The second coder (R2) reviewed the complete manuscripts to establish their own coding schemes and themes. The codes to be used were then discussed by each coder and the coded data were compared. Differences were discussed and the final coding was completed.

## Ethics

Ethical approval was provided by Flinders University’s Social and Behavioural Research Ethics Committee, project number 6719. Additional endorsements for the project were provided by the facilitating partner ac.care Communities for Children Murraylands.

## Results

Four main themes were identified from the data regarding the experiences of groups engaged with the Antenatal Dads and First Year Families program. The first theme was ‘Knowledge and Capacity Building’ and explored the father’s positive response to the information and services provided. The second theme was ‘Mental Health Awareness’, which identified the importance of raising awareness and early intervention regarding mental health as well as addressing how and where to get help. The third theme was ‘Soft-Entry’, which discussed how attendance at one service helped participants to learn about additional services through ‘word of mouth’ and targeted promotion. The final theme was ‘Feeling Connected’, which discussed how the program helped fathers to feel more connected with the process of child birth and with people sharing a similar situation to their own.

### Theme 1: Knowledge and capacity building

In many instances, there were positive comments about the comprehensive nature of the information provided during the program, and how this information helped to raise awareness within the fathers. This increased awareness in turn was identified by staff as helping otherwise ‘at risk’ fathers to build their capacity regarding (which crucially includes the acceptance of) their impending fatherhood. This raised awareness also indirectly helps build capacity for both the mother and the child by having the support of the engaged father to draw on.

One staff member provided an example of the programs content:

The antenatal program we talk about attachment theory, the importance of fathering, and the bonding that occurs. I hand around a model of an infant’s brain, 400 grams and then a 3 year old at 1200 grams. The dads are shocked at the growth. We give them [dads] an understanding of the importance of talking to by even in utero. They go on to talk about how they were fathered and their relationship with their fathers and how it needs to be different now. (D7)

Educating fathers on topics such as infants, parenting and having them reflect on their own childhood and the need to address deficits experienced when fathered by their fathers, helped fathers to better ‘understand’ and ‘attach to’ their infants/children. Participant D2 noted the sections of the education that stood out for them and of the impact this knowledge had:

We talked about all sorts of things…skin to skin contact for dad and baby, looking into baby’s eyes, breast feeding. It’s very good for us dads so we know, we can help. So much information it was really good. I felt comfortable asking questions. I feel I know a lot more now…you know what to expect. (D2)

Staff member C1 noted that the ‘program is unique, it focuses on the Dads and really gets them engaged into being a father’ and provided an example for why this was important as ‘The dads are often “at risk”, so they’re young, Indigenous, or poor, or CALD backgrounds and frankly without this program their kids would be at risk of removal’.

This comment highlights how the program indirectly helps to build capacity around the child by helping dads to engage with their child. Capacity building was also noted by fathers who discussed being connected to additional services if required (discussed in theme 3) or being made aware of other services that may help. This capacity raising is important as most fathers discussed their isolation from other families and services and how their participation in the program provided a means for them to connect back to the community.

### Theme 2: Mental health awareness

The topics of mental health and suicide were actively discussed in the Antenatal Dads and First Year Families program. Fathers noted being impressed by the manner in which ‘sensitive topics such as depression and suicide were addressed’. The prevalence of mental health issues for young fathers in a rural community surprised some participants as D1 and D4 state:

You don’t think about it much in dads. You think about it in the mums all the hormones and everything. It really opened my eyes and gave me lots to think about, you know how I could get help and stuff. They talk about that too all the help that’s out there. (D1)

We went through male depression…I was surprised that men get it too…extra information about where to go and to help us cope. All the information on depression, suicide in fathers all helps and the information on baby and mum helps me worry less. It was good to talk about these things [depression and suicide] without the women being present. (D4)

Participant D2 also picked up on D1 and D4 points regarding the importance of the program’s education on how and where to gain help, but also noted the importance of early intervention:

We need to know where to go to get help [for mental health issues] the Antenatal Dads stuff lets you know what to do and where to get help. You can bring stuff out in the open…get help early. Yes, the early warning signs…who to contact and that. (D2)

Also, of note in the above comments was D4’s remark on the importance (to him) of being able to discuss the sensitive subject of mental health and suicide without women present, which suggests in a mixed or partnered format, D4 may have remained quiet.

Critical incidents, such as suicide and depression, can have powerful impacts on small rural communities. The pertinences of a discussion about suicide within a young rural male cohort who were also socially disadvantaged were made sadly clear when participant D5 noted:

We’ve had four suicides here recently…all within six months and I knew them all so I already feel down in the dumps…we talked about going to the doctor and stuff or talk to someone. I’ve seen it with them they all hold it in…it has a roll on effect…having more knowledge and knowing what to expect helps you deal with it [depression]. (D5)

All fathers interviewed appeared to know the fathers who had committed suicide and the impact it had on their surviving spouses and children, which also seemed to re-enforce the importance of knowledge on the topic, and of how to get help. This point was well summed up by participant D3 who stated:

It was a bit confronting when they [the fatherhood worker] started talking about it [depression and suicide]. Oh shit you know- but it’s for the best. It might be confronting to start off with but it is for the best. I know what to look for and how to get help now. (D3)

Staff were also aware of the recent suicides in the area as C1 noted:

We have had some suicides lately in the community, it’s tough in a rural community and it is stressful having a child. The Antenatal Dads has really helped with all the mental health stuff. The dads and families are much happier and more confident afterwards. You can see the difference. (C1)

This change in outlook for those attending the antenatal dads program was also noted in participant data, for example, D5 notes:

I feel supported here. Its ok to talk about all sorts of stuff and the other men feel the same. You can talk about anything. We have made friends with other couples who are having the baby at the same time. I didn’t realise how I was worried, like really worried about what was going to happen. I don’t feel so alone you know worried. (D5)

This opportunity to openly discuss sensitive topics was expressed as a relief for the fathers interviewed who, as noted above, may not have otherwise talked in depth on the topic.

### Theme 3: Soft entry

The staff and fathers attending the program highlighted the importance of the ‘soft entry’ approach where attendance at one service helps people identify other services through word of mouth or through direct promotion by program staff. An example of the soft entry approach, and its importance to disadvantaged families, is explained by S1 and S2 as:

At the Antenatal Dads and First Year Families program I tell the dads about all the other programs they can access in the community. How they can go along…they don’t need a referral. They can attend. We are here to help each other…it’s a way of getting them connected to other services as well. (S1)

A lot of the dads have had no experience with infants and children…they don’t know what to do…some are young parents too and their infants can be neglected so it’s a good way to connect early and let them talk to the other dads and support each other. Antenatal Dads and First Year Families program lets them hear about other services they [fathers] can use…gives them confidence to use other services…helps them connect to other fathers and families. (S2)

The soft entry approach helps increase supports around the father by increasing their knowledge of the aid that is available to them, as explained by S1:

We use the Antenatal Dads and First Year Families program as a way of connecting clients with other services …providing those wrap around services. So the family support worker would provide case management, therapeutic support but also offer referrals to other agencies. (S1)

Interview data from the fathers helped understand the importance of this approach as fathers were either unaware of the service within the local area that were available to them, or of the ease with which those services could be accessed. As D3 relates:

You know, I came here and I didn’t realise just how many services we have in Murray Bridge. I never knew we could get so much help. You don’t need to go anyway to get a referral you can attend here it much easier…the other services you can go to don’t need referrals either. Referrals are such a hassle when you’ve got work, home and everything. (D3)

The concept of ‘soft entry’ initiatives is important as it helps connect services to disadvantaged and isolated families, and helps integrate these families and their children into the health, education and social systems provided by, or linked to these services.

### Theme 4: Feeling connected

The program provides fathers with age appropriate information about child development. This helped fathers to understand notions like why talking to their children while in utero was important to the child development, as the child was able to hear them and develop a bond with their voice. It also had the unintended side effect of having fathers feel more connected to their children, as explained by D5:

I know to talk to baby, you know, while it’s in my wife’s tummy…I didn’t know that was important…it has helped me feel connected. I didn’t feel that before…I didn’t know about what was happening inside my wife and how the baby can hear. I know what to do now. You can read to baby right from the beginning you don’t need to wait. (D5)

Throughout the results are the examples of fathers discussing how the knowledge gained from the program had helped them to feel less worried. For example, D2 noted how his stress had been reduced due to participation in the program:

I stress a bit and if I don’t know then it’s hard, now I know what to expect, [attendance was] most definitely worthwhile. (D2)

Participants also spoke of making connections with other fathers (and even other couples who were expecting) that, without attendance at the program, would not have occurred. For example, D3 states:

[the program staff member] puts you in the middle [of the group] and talks about what’s around…how you connect and talk to others…other dads. You feel connected…I talk to other dads there and that wouldn’t happen without this program. (D3)

As D5 notes in theme 2, the connection that developed with other fathers through attendance at the Antenatal Dads and First Year Families’ program helped participants feel more connected and hence, less alone.

## Discussions

Between 2010 and 2016, the Antenatal Dads and First Year Families program assisted more than 328 fathers and their families. The program has developed a reputation for assisting fathers to develop and maintain positive and productive relationships with their children through improved knowledge and understanding. Overall, the fathers were representative of the broader regional population, as Aboriginal men, CALD fathers, farmers and a mixture of first-time and experienced fathers took part. All participants, regardless of designation, acknowledged that the Antenatal Dads and First Year Families programs provided a ‘safe’ and ‘supported’ space for men to acknowledge their fears and concerns about parenting and supporting their partners. Specifically of note on this topic was fathers’ feeling able to discuss sensitive subjects such as mental health and suicide when women were not present. The findings of this study suggest that for some men, male-only programs might offer a space where they feel more comfortable to open up. This in turn allows men to discuss sensitive topics that, in different circumstances, might remain untouched as found in Darwin *et al.*’s (2017) research where fathers reported a reluctance to acknowledge or discuss mental health issues. However, given the limited knowledge regarding men’s participation in parenting classes (Bronte-Tinkew *et al.*, 2007; Fletcher *et al.*, 2014; Scourfield, 2014), considerably more research is needed on the topic.

Multiple articles (Tully, 2009; Richter & Naicker, 2013; Borrelli *et al.*, 2015; Love *et al.*, 2016; Parry & Abbott, 2018) discussed how parenting programs reduced stress in parents, which provided a flow on the effect of producing more favourable environments for the child. Baldwin *et al.* (2018) and Burgess and Goldman’s (2018) studies noted that fathers experienced stress and mental health like symptoms both before, and after the birth of their child indicating that the event can have significant mental health implications for fathers. Despite this, no article found specifically reviewed the effects of mental health education on the fathers. Fathers, as discussed, expressed being able to be more open regarding sensitive topics such as mental health in male-only company, but also related feeling less worried and having greater knowledge of mental health because of the programs focus on the topic. This increased knowledge included an awareness of the warning signs regarding different mental health issues and an increased understanding of how and where to gain help. The pertinence of this type of education cannot be understated for disadvantaged fathers, in a rural setting, who are about to undergo a major life change. As the fathers in this study themselves noted, four fathers in the local community had recently committed suicide. Furthermore, for many, the knowledge gained from attendance in the program, especially on the topic of mental health and suicide, seemed to unlock something deeper in participants. It is noted in several responses that new knowledge gained from attendance at the program had helped participants identify a sub-conscious worry, stress or anxiety regarding their impending life change. This new understanding also appeared to aid participants to embrace the coming change, which was also observed by the program staff.

Several authors note that parenting classes dedicated to, or that include fathers in the education processes, have helped to change fathers’ beliefs about parenting and engage them more in the postnatal period (Bronte-Tinkew *et al.*, 2007; Tully, 2009; Fletcher *et al.*, 2014; Panter-Brick *et al.*, 2014; Scourfield, 2014). This, in turn, is suggested as benefitting the longer term outcomes for the child (Bronte-Tinkew *et al.*, 2007; Tully, 2009; Fletcher *et al.*, 2014; Panter-Brick *et al.*, 2014; Scourfield, 2014). However, no study appears to identify the specific mechanism that aids these changes in the father. Some insights identified in previous articles (Bronte-Tinkew *et al.*, 2007; Fletcher *et al.*, 2014; Scourfield, 2014) that are shared in this study suggest that antenatal education helps fathers to understand pregnancy and child development, which reduces anxiety, stress and disengagement through increased insight about the process. This study adds the knowledge that fathers meeting other fathers through attendance in the program, or even other couples in similar situations, helped fathers to feel less alone regarding their situation.

The strengthening of the father’s outlook through education is further increased through the promotion and/or referral to on-services that were either actively promoted by program staff, or passed between fathers through word of mouth. This is encouraging as literature (Bronte-Tinkew *et al.*, 2007; Ferrell, 2013; Zanoni *et al.*, 2013; Panter-Brick *et al.*, 2014; Scourfield, 2014; Baldwin *et al.*, 2018; Parry & Abbott, 2018) suggests that fathers faced considerable barriers to attending services and even reported feeling left out of parenting education opportunities. Fathers in this study reported that they encouraged other fathers to attend the services, which helps break down some of the barriers that restrict father’s attendance at service. Equally, having a place where fathers can find out about and gain referrals to other services also helps combat these noted barriers and again hopefully makes fathers feel more engaged. The building of supports around the father as well as increasing their antenatal, parenting and mental health knowledge all address aspects of the SDH. This strengthening of the father also potentially benefits both the mother and child as the father is better able to support them, when he is more supported in addressing his own concerns. Finally, participants also noted the need for the program to continue and to help improve the circumstances of new families against the potential harms caused by the SDH linked to disadvantage (Wilkinson *et al.*, 2009; Maggi *et al.*, 2010; Solar & Irwin, 2010; Shonkoff and Garner^2012^; Pickett & Wilkinson, 2015; Keleher, 2017).

Recommendation for new research includes an investigation into the extent to which men are more likely to open up in male-only programs, and if this consideration holds significance over couples, or mixed programs. Programs and research that separate results by gender will also aid understanding in the area (Zanoni *et al.*, 2013; Fletcher *et al.*, 2014; Panter-Brick *et al.*, 2014). Regardless, the results do offer some recommendation for practice such as support for the existence of male-only programs and potentially an insight into how to reach out to reluctant fathers. To support reluctant fathers, future research should consider how best to approach the subject of mental health so that education on the topic can reach as many people as possible. This includes a consideration for the language used in describing mental health and how best to help fathers identify and relate with the symptoms. As Baldwin *et al.* (2018) and Darwin *et al.* (2017) identify, men’s accounts of mental health may not fit clinical descriptions suggesting that the language used when discussing mental health may need adjusting to better fit the intended audience.

Identifying the language that men use in relation to mental health will also help program operators to identify fathers who might be at risk regarding their mental health. Moreover, the addition of mental health education in general to disadvantaged men, especially in settings of increased risk of male suicide such as rural/remote Australia, should be required content for any adult education program. This statement reflects the devastating stories of four local suicides related by the men in this study. Another recommendation for future research regards a consideration of the education content delivered in these programs and what specifically helps men to (or not to) re-engage with parenthood, is there a universal message that can be applied across programs? Or is it better to tailor material to the intended group? This also includes a consideration of the referrals to on-services that are recommended to fathers.

## Limitation

Demographic information about the fathers who took part in the research was restricted due to ethical concerns regarding their vulnerable status (e.g. material deprivation) as well as confidentiality concerns as all participants lived in the same rural area. Moreover, there was a general reluctance in participants to share demographic data; hence, additional information regarding the cohort cannot be given. While acting to protect those who took part in the research, it is an acknowledged limitation as it inhibits the generalisability of the study. Furthermore, being a qualitative study with a small sample, limited to participants sourced from one geographical location means that the results cannot be confidently generalised to larger populations. However, the study does provide useful insight into father’s experiences of parenting classes, in disadvantaged cohorts, which is limited within the current literature. Finally, the data collection was conducted by female members of the research team only, and this does indicate a gender bias in the analysis. However, the fathers did state that they were happy to participate with data collected by an all-female team.

## Conclusion

The success of the Antenatal Dads and First Year Families program is reflected in the positive attitudes of fathers involved as discussed within the themes section presented above. This should hopefully provide motivation to service provider to consider male-only antenatal and/parenting classes. However, the best format for these classes, their implementation, and how to engage more men from disadvantaged groups still needs further investigation. It is encouraging to note that fathers, through engagement with the program, developed further social and support networks that positively assist their development as fathers. This, as well as linking fathers (and so theoretically their families also) to other community services, will further help these families to combat the SDH going forward.

## Footnotes

1 A program delivered to disadvantaged African-American children in the 1960’s.

2 A program delivered to disadvantaged children up to 9 years of age and their families in Chicago public schools.

## References

[ref51] AEDC (2015) *Data explorer: South Australia* Retrieved from https://www.aedc.gov.au/data/data-explorer

[ref1] Baldwin S, Malone M, Sandall J and Bick D (2018) Mental health and wellbeing during the transition to fatherhood: a systematic review of first time fathers’ experiences. JBI Database of Systematic Reviews and Implementation Reports 16, 2118–2219. doi: 10.11124/JBISRIR-2017-003773 30289768 PMC6259734

[ref2] Bartik TJ (2011) Investing in kids: early childhood programs and local economic development. Michigan, USA: WE Upjohn Institute for Employment Research.

[ref3] Baum F (2011) The New Public Health, third edition South Melbourne, Victoria: Oxford Uinversity Press.

[ref4] Belfield C, Nores M, Barnett S and Schweinhart L (2006) The High/Scope Perry preschool program: cost-benefit analysis using data from the age-40 followup. The Journal of Human Resources 41, 162–190.

[ref52] Borrelli B, Tooley EM and Scott-Sheldon LA (2015) Motivational interviewing for parent-child health interventions: a systematic review and meta-analysis. Pediatric Dentistry 37, 254–265. Retrieved from https://www.ncbi.nlm.nih.gov/pubmed/26063554 26063554

[ref5] Bowes J and Grace R (2014) Review of early childhood parenting, education and health intervention programs for Indigenous children and families in Australia. Issues paper no. 8. *Closing the Gap Clearinghouse* Canberra: Australian Institute of Health and Welfare and Melbourne, Australian Institute of Family Studies.

[ref50] Bronte-Tinkew J, Ryan S, Carrano Jand Moore K (2007) Resident fathers’ pregnancy intentions, prenatal behaviors, and links to involvement with infants. Journal of Marriage and Family 69, 977–990.

[ref6] Burgess A and Goldman R (2018) Who’s the bloke in the room? Fathers during pregnancy and at the birth in the United Kingdom. United Kingdom: Fatherhood Institute and Nuffield Foundation.

[ref7] Cavana RY, Delahaye BL and Sekaran U (2001) Applied business research: qualitative and quantitative methods. Queensland, Australia: John Wiley and Sons Australia.

[ref8] Coren E, Hossain R, Pardo Pardo J, Veras MM, Chakraborty K, Harris H and Martin AJ (2013) Interventions for promoting reintegration and reducing harmful behaviour and lifestyles in street-connected children and young people. Cochrane Database of Systematic Reviews, Issue 2, CD009823. doi: 10.1002/14651858.CD009823.pub2 23450609

[ref9] Darwin Z, Galdas P, Hinchliff S, Littlewood E, McMillan D, McGowan L and Gilbody S (2017) Fathers’ views and experiences of their own mental health during pregnancy and the first postnatal year: a qualitative interview study of men participating in the UK Born and Bred in Yorkshire (BaBY) cohort. BMC Pregnancy and Childbirth 17, 1229–1244. doi: 10.1186/s12884-017-1229-4 PMC5270346 28125983

[ref10] Dockery AM, Kendall G, Li J, Mahendran A, Ong R and Strazdins L (2010) Housing and children’s development and wellbeing: a scoping study. Melbourne, Australia: Australian Housing and Urban Research Institute.

[ref11] Embleton L, Mwangi A, Vreeman R, Ayuku D and Braitstein P (2013) The epidemiology of substance use among street children in resource-constrained settings: a systematic review and meta-analysis. Addiction 108, 1722–1733. doi: 10.1111/add.12252 23844822 PMC3776018

[ref53] Englund MM, White B, Reynolds AJ, Schweinhart L and Campbell FA (2015) Health Outcomes of the Abecedarian, Child-Parent Center, and HighScope Perry Preschool Programs In Reynolds AI, Rolnick AT and Temple JA, editors. Health and Education in Early Childhood: Predictors, Interventions, and Policies. Cambridge, UK: Cambridge University Press, 257–292.

[ref54] Ferrell K (2013) *Where Are All the Dads?: Exploring the Barriers to Engaging Fathers in Child Protective Services Cases and the Strategies to Overcoming the Barriers*. Texas State University - San Marcos: Unpublished doctoral thesis.

[ref55] Fletcher R, May C, St George J, Stoker L and Oshan M (2014) Engaging fathers: evidence review. ACT, Australia: Australian Research Alliance for Children and Youth.

[ref12] Gibson C and Johnstone T (2010) Investing in our future: children’s journey’s through homelessness and child protection: a scan of the literature, policy and practice. Adelaide, South Australia: Australian Cente for Child Protection.

[ref56] Glaser B and Strauss A (1967) The discovery of grounded theory: strategies for qualitative research. Oxon, UK: Routledge.

[ref13] Green J and Thorogood N (2014) Qualitative methods for health research, third edition London, UK: Sage Publications.

[ref14] Hansen E (2006) Successful qualitative health research: a practical guide. New South Wales, Australia: Allen and Unwin.

[ref15] Havighurst S, Kehoe C, Harley A and Wilson K (2015) Tuning in to Kids: an emotion-focused parenting intervention for children with disruptive behaviour problems In Essau C and Allen J, editors, Making parenting work for children’s mental health. London, UK: Association for Child and Adolescent Mental Health, Occasional papers 33 (chap 5, 41–50)

[ref16] Havighurst S, Wilson K, Harley A and Prior M (2009) Turning in to kids: an emotion focused parenting program: intitial findings from a community trial. Journal of Community Psychology 37, 1008–1023.

[ref57] Heckman JJ, Moon SH, Pinto R, Savelyev PA and Yavitz A (2010) The rate of return to the HighScope Perry Preschool Program. Journal of Public Economics 94, 114–128. 21804653 10.1016/j.jpubeco.2009.11.001 PMC3145373

[ref17] Hunter C and Meredith V (2015) The utility of a reflective parenting program for parents with complex needs: an evaluation of Bringing Up Great Kids. Ringwood, Victoria, Australia: Australian Government – Australian Instution of Family Studies and the Australian Childhood Foundation.

[ref60] Keleher H (2017) Health education and empowerment In Keleher H and MacDougall C, editors, Understanding Health, fourth edition South Melbourne, Victoria: Oxford University Press, 254–269.

[ref18] Keleher H and MacDougall C (2017) Determinants of Health In Keleher H and MacDougall C, editors, Understanding Health, fourth edition South Melbourne, Victoria: Oxford University Press, 19–34.

[ref19] Keys D (2009) Children and homelessness: literature review. Blackburn, Victoria: The Salvation Army Australia Southern Territory, Melbourne.

[ref20] Kilmer R, Cook J, Crusto C, Strater K and Haber M (2012) Understanding the ecology and development of children and families experiencing homelessness: implications for practice, supportive services, and policy. American Journal of Orthopsychiatry 82, 389–401. doi: 10.1111/j.1939-0025.2012.01160.x 22880977

[ref49] Kraus N, Hornickel J, Strait DL, Slater J and Thompson E (2014) Engagement in community music classes sparks neuroplasticity and language development in children from disadvantaged backgrounds. Frontiers in Psychology 5, 1403. 25566109 10.3389/fpsyg.2014.01403 PMC4268440

[ref21] Kuehn BM (2014) AAP: toxic stress threatens kids’ long-term health. JAMA 312, 585–586. doi: 10.1001/jama.2014.8737 25075798

[ref62] Love SM, Sanders MR, Turner KM, Maurange M, Knott T, Prinz R, Metzler C and Ainsworth AT (2016) Social media and gamification: engaging vulnerable parents in an online evidence-based parenting program. Child Abuse & Neglect 53, 95–107. doi: 10.1016/j.chiabu.2015.10.031. 26880281

[ref22] Lynam M, Loock C, Scott L, Wong S, Munroe V, Palmer B, Fitzsimmons B and Fitzgerald B (2010) Social pediatrics initiative: enacting a ‘RICHER’ model (Responsive Intersectoral and Interdisciplinary Child Health Education and Research). British Colombia, Canada: British Colombia Medical Services Foundation and Canadian Nurses Foundation.

[ref23] MacDougall C (2017) Determinants of Behaviours In Keleher H and MacDougall C, editors, Understanding Health, fourth edition South Melbourne, Victoria: Oxford University Press, 155–175.

[ref24] Maggi S, Irwin LJ, Siddiqi A and Hertzman C (2010) The social determinants of early child development: an overview. The Journal of Paediatrics and Child Health 46, 627–635. doi: 10.1111/j.1440-1754.2010.01817.x 20796183

[ref25] Marmot M (2006) Introduction In Marmot M and Wilkinson RG, editors, Social Determinants of Health, second edition Oxford, United Kingdom: Oxford University Press.

[ref26] McCoy-Roth M, Mackintosh B and Murphey D (2012) When the bough breaks: the effects of homelessness on young children. Child Trends 3, 1–11.

[ref27] Moffitt TE, Arseneault L, Belsky D, Dickson N, Hancox RJ, Harrington H, Houts R, Poulton R, Roberts B, Ross S, Sears M, Thomson W and Caspi A (2011) A gradient of childhood self-control predicts health, wealth, and public safety. Proceedings of the National Academy of Sciences of the United States of America 108, 2693–2698. doi: 10.1073/pnas.1010076108. 21262822 PMC3041102

[ref28] Muir K, Katz I, Edwards B, Gray M, Wise S, Hayes A and Community Strategy evaluation team (2010) The national evaluation of the communities for children initiative. Family Matters 84, 35–42.

[ref29] Mustard J (2006) Experience-based brain development: scientific underpinnings of the importance of early child development in a global world. Paediatrics and Child Health 11, 71–572. doi: 10.1093/pch/11.9.571. PMC2528649 19030325

[ref30] Mustard J (2010) Preface In Bammer G, Michaux A and Sanson A, editiors, Bridging the ‘know-do’ gap: knowledge brokering to improve child wellbeing. Canberra, Australia: Australian National University E-Press.

[ref31] Nelson F and Mann T (2011) Opportunities in public policy to support infant and early childhood mental health: the role of psychologists and policymakers. American Psychologist, 66, 129–139. doi: 10.1037/a0021314. 21142338

[ref32] Noble KG, Norman MF and Farah MJ (2005) Neurocognitive correlates of socioeconomic status in kindergarten children. Developmental Science 8, 74–87. doi: 10.1111/j.1467-7687.2005.00394.x. 15647068

[ref33] Noble-Carr D (2007) The experiences and effects of family homelessness for children: a literature review. Canberra, Australia: Institute of Child Protection Studies.

[ref58] Nyberg G, Norman A, Sunbolm E, Zeebari Z and Elinder L (2016) Effectiveness of a universal parental support programme to promote health behaviours and prevent overweight and obesity in 6-year-old children in disadvantaged areas, the Healthy School Start Study II, a cluster-randomised controlled trial. International Journal of Behavioural Nutrition and Physical Activity 13, 1–14. doi: 10.1186/s12966-016-0327-4. PMC4721005 26795378

[ref59] Panter-Brick C, Burgess A, Eggerman M, McAllister F, Pruett K and Leckman JF (2014) Practitioner review: engaging fathers–recommendations for a game change in parenting interventions based on a systematic review of the global evidence. Journal of Child Psychology and Psychiatry 55, 1187–1212. 24980187 10.1111/jcpp.12280 PMC4277854

[ref34] Parker R (2009a) Hey dad! For indigenous dads, uncles and pops. Family Relationships Quarterly 12, 13–14.

[ref35] Parker R (2009b) Turning in to kids: emotionally intelligent parenting. Family Relationships Quarterly 12, 15–17.

[ref36] Parry YK and Abbott S (2018) Independent evaluation: Being with baby program (final report). Bedford Park, South Australia: Flinders University, Salvation Army Salisbury and Lutheran Community Care SA.

[ref37] Patton M (2015) Qualitative research and evaluative methods, fourth edition London, UK: Sage Publications.

[ref61] Pickett KE and Wilkinson RG (2015) Income inequality and health: a causal review. Social Science & Medicine 128, 316–326. doi: 10.1016/j.socscimed.2014.12.031. 25577953

[ref38] Reynolds AJ, Temple JA, White BA, Ou SR and Robertson DL (2011) Age 26 cost-benefit analysis of the child-parent center early education program. Child Development 82, 379–404. doi: 10.1111/j.1467-8624.2010.01563.x. 21291448 PMC3817956

[ref39] Richter L and Naicker S (2013) A review of published literature on supporting and strengthening child- caregiver relationships (parenting). Virginia, USA: AIDSTAR-One.

[ref40] Roos LE, Mota N, Afifi TO, Katz LY, Distasio J and Sareen J (2013) Relationship between adverse childhood experiences and homelessness and the impact of axis I and II disorders. American Journal of Public Health 103 (Suppl 2), S275–S281. doi: 10.2105/AJPH.2013.301323. 24148049 PMC3969113

[ref41] Rossiter C, Power T, Fowler C, Jackson D, Roche M and Dawson A (2017) Learning to become a better man: insights from a fathering programme for incarcerated Indigenous men. Austustraian Journal of Social Issues 52, 13–31. doi: 10.1002/ajs4.4.

[ref63] Scourfield J (2014) Improving work with fathers to prevent child maltreatment: fathers should be engaged as allies in child abuse and neglect prevention. Child Abuse & Neglect 38, 974–981. doi: 10.1016/j.chiabu.2014.05.002. 24873732

[ref64] Shonkoff JP and Garner AS (2012) The lifelong effects of early childhood adversity and toxic stress. Pediatrics 129, e232–e246. 22201156 10.1542/peds.2011-2663

[ref42] Solar O and Irwin A (2010) A conceptual framework for action on the Social Determinants of Health. Geneva, Switzerland: World Health Organisation.

[ref44] Taylor P, Moore P, Pezzullo L, Tucci J, Goddard C and De Bortoli L (2008) The cost of Child Abuse in Australia. Melbourne, Victoria: Access Economics, Australian Childhood Foundation and Child Abuse Prevention Research Australia at Monash University.

[ref45] Tong A, Sainsbury P and Craig J (2007) Consolidated criteria for reporting qualitative research (COREQ): a 32-item checklist for interviews and focus groups. International Journal for Quality in Health Care 19, 349–357. 17872937 10.1093/intqhc/mzm042

[ref46] Tully L (2009) What makes parenting programs effective? An overview of recent research. New South Wales, Australia: NSW Department of Community Services.

[ref47] Wadsworth M and Butterworth S (2006) Early life In Marmot M and Wilkinson RG, editors, Social Determinants of Health, second edition Oxford, United Kingdom: Oxford University Press, 31–53.

[ref65] Wilkinson R, Pickett K and Cato MS (2009) The spirit level. Why more equal societies almost always do better. New York, USA: Penguin Books.

[ref66] Zanoni L, Warburton W, Bussey K and McMaugh A (2013) Fathers as ‘core business’ in child welfare practice and research: an interdisciplinary review. Children and Youth Services Review 35, 1055–1070. 10.1016/j.childyouth.2013.04.018.

[ref48] Zlotnick C, Tam T and Zerger S (2012) Common needs but divergent interventions for U.S. homeless and foster care children: results from a systematic review. Health and Social Care in the Community 20, 449–476. doi:10.1111/j.1365-2524.2011.01053.x. 22356430

